# The Role of Base Excision Repair in Major Depressive Disorder and Bipolar Disorder

**DOI:** 10.1192/j.eurpsy.2022.1016

**Published:** 2022-09-01

**Authors:** M. Kucuker, A. Ozerdem, D. Ceylan, A. Cabello-Arreola, M.C. Ho, B. Joseph, L. Webb, P. Croarkin, M. Frye, M. Veldic

**Affiliations:** 1 Mayo Clinic Depression Center, Department Of Psychiatry & Psychology, Rochester, United States of America; 2 Koc University, Department Of Psychiatry & Psychology, Istanbul, Turkey; 3 Mayo Clinic Depression Center, Department Of Psychiatry & Psychology, Scottsdale, United States of America; 4 Mayo Clinic, Alix School Of Medicine, rochester, United States of America

**Keywords:** major depressive disorder, Oxidative damage, DNA repair, base excision repair

## Abstract

**Introduction:**

In vivo and in vitro studies suggest that inflammation and oxidative damage may contribute to the pathogenesis of major depressive disorder (MDD) and bipolar disorder (BD). Imbalance between DNA damage and repair is an emerging research area examining pathophysiological mechanisms of these major mood disorders.

**Objectives:**

This systematic review sought to examine current evidence on the association between mood disorders and deficits in base excision repair (BER), the primary repair mechanism for repair of oxidation-induced DNA lesions.

**Methods:**

We conducted a comprehensive literature search of Ovid MEDLINE® Epub Ahead of Print, Ovid MEDLINE® In-Process & Other Non-Indexed Citations, Ovid MEDLINE® Daily, EMBASE (1947), and PsycINFO for studies investigating the alterations in base excision repair in patients with MDD or BD.

**Results:**

A total of 1,364 records were identified. 1,352 records remained after duplicates were removed. 24 records were selected for full-text screening and a remaining 12 articles were included in the qualitative synthesis. SNPs (Single Nucleotide Polymorphisms) of several BER genes have been shown to be associated with MDD and BD. However, it was difficult to draw conclusions from BER gene expression studies due to conflicting findings and the small number of studies.

**Conclusions:**

Future studies comparing DNA repair during the manic or depressive episode to remission will give us a better insight regarding the role of DNA repair in mood disorders. These alterations might be utilized as diagnostic and prognostic biomarkers as well as measuring treatment response.

**Disclosure:**

No significant relationships.

